# Thoracic SMARCA4-Deficient Undifferentiated Tumor in a 40-Year-Old Male Welder With No Smoking History

**DOI:** 10.7759/cureus.76203

**Published:** 2024-12-22

**Authors:** Samuel MacDowell, Mufadda Hasan, Ahmad Ibrahim

**Affiliations:** 1 Medical Education, California University of Science and Medicine, Rialto, USA; 2 Pulmonary and Critical Care Medicine, Arrowhead Regional Medical Center, Colton, USA; 3 Pathology and Laboratory Medicine, Arrowhead Regional Medical Center, Colton, USA

**Keywords:** atezolizumab, etoposide and cisplatin, immuno-histochemical, non-small cell lung cancer (nsclc), non-smoker lung cancer, pancoast, smarca4-deficient, tsdut, vertebral metastasis, welding

## Abstract

Thoracic SMARCA4-deficient undifferentiated tumor (TSDUT) is a rare and quite new classification of primary pulmonary malignancy. It is classified as a non-small cell lung cancer, typically associated with smoking, and is highly aggressive. Its clinical features, immunohistochemistry, and pathology are quite unique.

In this case report, we describe the clinical course of TSDUT pancoast tumor in a 40-year-old male, without a substantial smoking history, but a significant history of occupational welding.

## Introduction

Thoracic SMARCA4-deficient undifferentiated tumor (TSDUT) is a rare and quite new classification of primary pulmonary malignancy. "It is officially recognized in the fifth edition of the World Health Organization classification of thoracic tumors as distinct from conventional non-small cell lung cancer, due to its unique morphology, immunohistochemical profile, and clinical presentation [1]. It has a significant association with substantial smoking history and occurs primarily in men with a median age of 48. The cancer is known to be highly invasive, aggressive, and unfortunately carries a poor prognosis [2]. Occasionally surgery may be pursued. Chemotherapy and immunotherapy are unclear as efficacious treatment modalities, though most results have not improved survival substantially [3].

Typically tumor morphology demonstrates incohesive rhabdoid tumor cells, with regional necrosis. Immunohistochemistry typically stains positive for synaptophysin, CD34, SOX2, and SALL4, though it is not as specific. Loss of the surrogate marker for SMARCA4, BRG1, is nearly always present [3,4].

In this case report, we describe the clinical course of TSDUT in a 40-year-old male, without a substantial smoking history, but a significant history of occupational welding.

## Case presentation

A 40-year-old Hispanic male welder with no significant smoking history, family history of lung cancer, or medical history other than a right-sided nephrectomy following a motor vehicle accident more than 20 years ago, presented to our emergency room in September 2023 after a right apical lung mass, identified at another facility in March 2023, was found. The patient was unable to complete the follow-up in March.

His initial symptoms in March were shortness of breath and vague chest pain. Since March, he noted the development of back pain, which progressively worsened by July 2023. In the week prior to his visit, he also experienced new right-sided tingling along the medial aspect of the right arm, along with numbness in the fourth and fifth digits. He was unaware of any weight loss. Over time, the back pain and paresthesia progressively interrupted more of his day-to-day activities.

**Figure 1:**
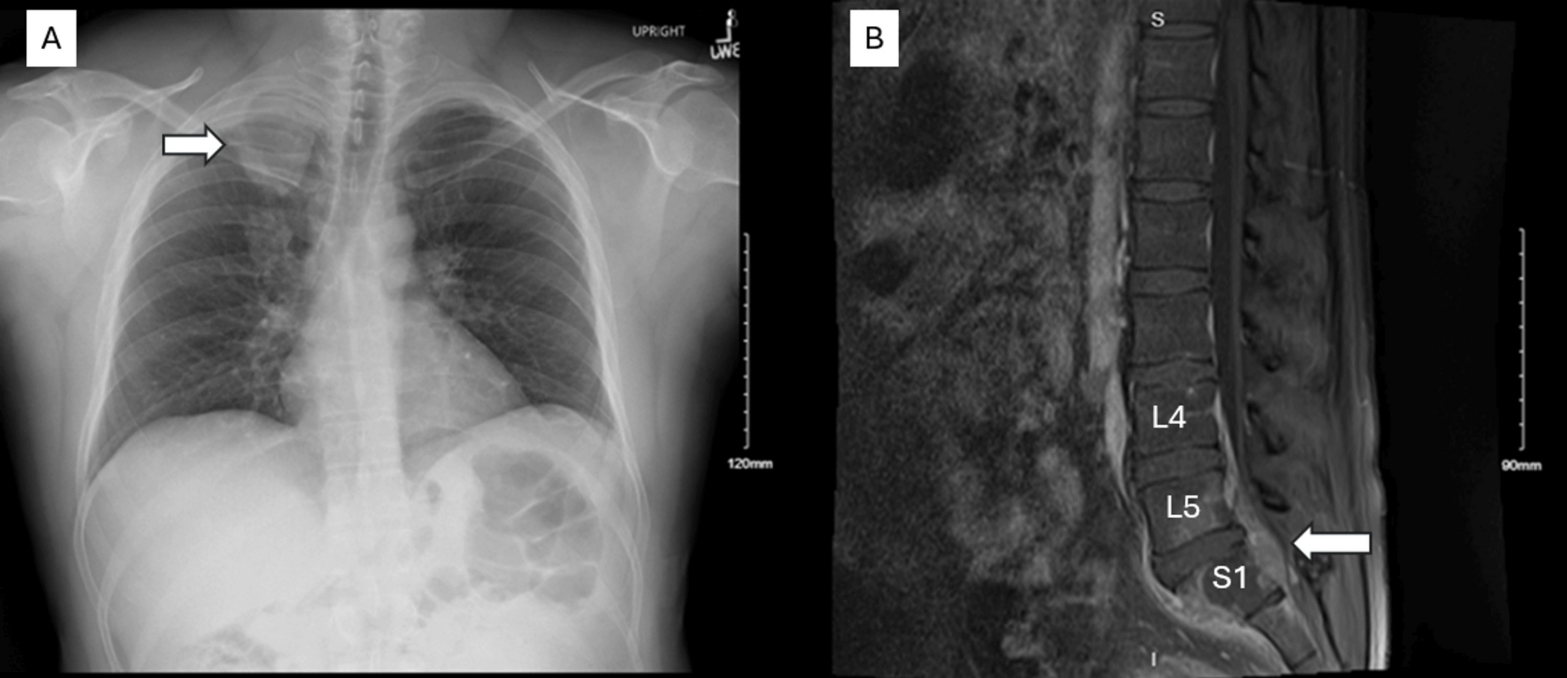
A) Upright chest X-ray on presentation, showing a right apical mass (arrow). B) Lumbar MRI with contrast (T1-weighted), showing significant stenosis at L5-S1 (arrow).

X-ray showed a large apical mass (Figure 1A). MRI scan demonstrated tumor involvement in L5 and S1, with a 12 mm component at S1 (Figure 1B).

**Figure 2:**
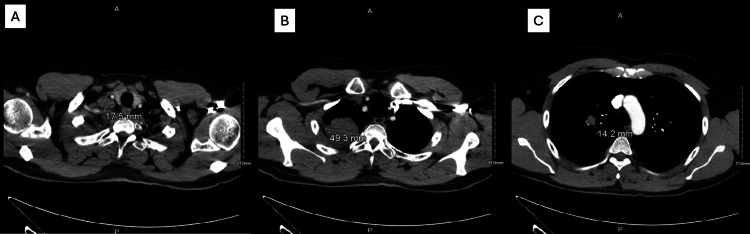
CT scan with IV contrast showing A) enlarged right supraclavicular lymph node, B) large right apical lung mass, and C) enlarged hilar masses.

CT scan with IV contrast demonstrated a right-sided 4.9 cm transverse soft tissue apical lung mass (Figure 2B). Multiple right hilar masses were present, measuring 1.4 cm in diameter, and a right supraclavicular lymph node measuring 1.7 cm in diameter (Figure 2A).

**Figure 3:**
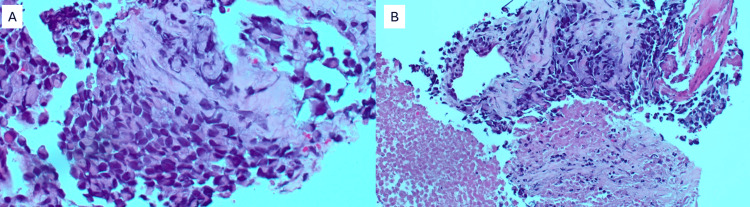
(A) Tumor fragments with rhabdoid morphology and necrosis at 20x magnification. (B) Eosinophilic vacuoles at 40x magnification.

**Figure 4:**
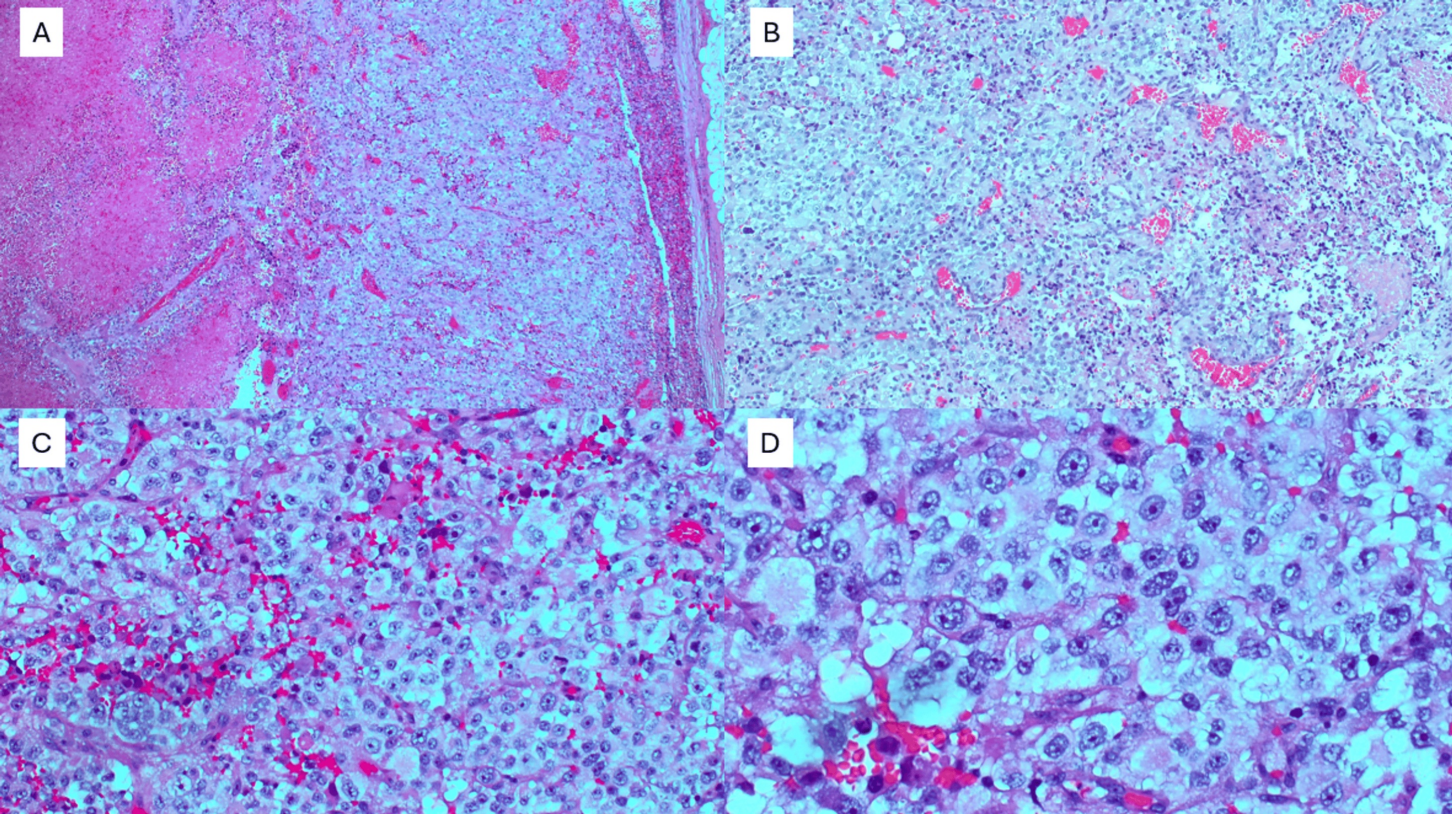
(A) Diffuse sheets of round and epithelioid cells with necrosis at 2x magnification and B) at 10x magnification. (C) Epithelioid cells exhibiting vesicular chromatin and prominent nuclei at 20x magnification. (D) Prominent, relatively monotonous nuclei at 40x magnification.

**Figure 5:**
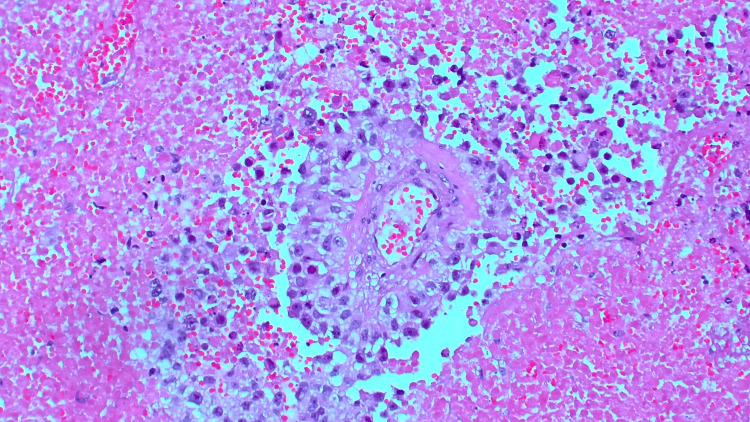
Occasionally, a peritheliomatous growth pattern with necrosis and mitotic figures can be seen in the excised biopsy.

Incisional biopsy of the inguinal lymph node demonstrated no malignancy; however, pathology from initial incisional supraclavicular biopsy demonstrated fragmentation of tumor cells with rhabdoid morphology, necrosis, and eosinophilic vacuoles (Figure 3A). Excisional lymph node gross examination showed variable discohesive sheets of epithelioid cells with regions of necrosis (Figure 4A) and occasional peritheliomatous growth patterns (Figure 5).

Immunohistochemistry staining reported positivity for Ki67, synaptophysin, CD56, OSCAR, vimentin, FLI-1, INSM1, chromogranin, and CAM5. Negative staining was reported for CKPan, CK7, CK20, CD45, CD30, CD99, Oct-3/4, desmin, myogenin, CD31, claudin-4, and ERG. Lesion cells were found to have lost BRG-1 (SMARCA4) and BRM (SMARCA2), but INI (SMARCB1) was preserved. Overall, the presence of rhabdoid tumor cells and neuroendocrine markers, specifically synaptophysin, along with the loss of BRG1, supports a diagnosis consistent with TSDUT. Cytopathological samples were reviewed by two cytopathologists including a specialist in thoracic tumors.

The patient received multiple cycles of cisplatin/etoposide and additional atezolizumab maintenance therapy. He received multiple cycles of palliative radiation to L5-S1 over the year. The patient developed neutropenic fever and an abscess in the right inguinal region as a complication. Later, the patient became bedbound due to the tumor’s progression and developed a pathologic fracture of the right femoral neck. A PET scan the following year demonstrated hypermetabolic destruction of the spinous processes of T5-T7. Despite initial improvement in lower extremity symptoms following radiation therapy, the patient eventually developed saddle anesthesia. MRI showed increasing enhancement of the S1 epidural lesion, with compression of the left and displacement of the right nerve root. After a year and a half since the discovery of the lesion, and a year following appropriate staging, the patient was placed on hospice care.

## Discussion

TSDUT is a rare and aggressive lung tumor that can occasionally present in non-smokers, often affecting relatively young males. One study found that approximately 12% of cases may occur in individuals without a smoking history [3]. Certain occupations with exposure to volatile oxidized metals, such as welding, have been shown to have a causal link to small cell and non-small cell lung cancers [5-7]. Welding fumes are considered a Group 1 carcinogen by the IARC [8]. To our knowledge, this is the first recorded instance of a non-smoker with significant occupational exposure developing TSDUT.

No treatment protocol has been shown to demonstrate efficacious remission in this disease, and most cases are discovered at an advanced stage. Recently, however, a PD-L1-negative TSDUT was shown to have a reduction in tumor burden following second-line treatment with tislelizumab in combination with etoposide and carboplatin, after failure of first-line treatment with four cycles of liposomal paclitaxel, cisplatin, and anlotinib [9]. In another case, a reduction in tumor burden was seen with conversion surgery following neoadjuvant therapy with bevacizumab, paclitaxel, and carboplatin [10]. In this case, a similar treatment protocol was followed, however, with atezolizumab to induce a PD-L1 blockade, which did not reduce tumor burden enough for further intervention.

This case also demonstrated that TSDUT can present as a Pancoast tumor with clinical characteristics more typical of non-small cell lung cancer rather than small cell lung cancer. Due to the right apex location, compression of the brachial plexus also may be responsible for the ulnar paresthesia, which is not typical of most small cell lung cancer or other cases described of TSDUT. The most common sites of primary malignancy are the left upper lobe and the mediastinum. Often, symptoms from the tumor compressing the pleura and lung parenchyma are reported, and distant metastasis can include the axial skeleton [3]. Axial skeletal involvement is prominent in this case due to L5-S1 lumbar stenosis from mass effect. Once again, a key clinical feature distinguishing non-small cell lung cancer from small cell lung cancer is the preference for bone metastasis over metastasis to solid organs [11].

## Conclusions

Given this case presentation, it is possible that TSDUT may have other associations other than smoking. It may also present as classical non-small cell lung apex tumors.
